# Analysis of Compliance and Kinetostatic of a Novel Class of *n*-4R Compliant Parallel Micro Pointing Mechanism

**DOI:** 10.3390/mi13071014

**Published:** 2022-06-27

**Authors:** Jun Ren, Qiliang Li

**Affiliations:** School of Mechanical Engineering, Hubei University of Technology, Wuhan 430068, China; 102000004@hbut.edu.cn

**Keywords:** compliant mechanism, pointing mechanism, flexure hinge, compliance matrix, kinetostatic

## Abstract

A novel class of *n*-4R compliant parallel pointing mechanisms is proposed, and the compliance and kinetostatic model of the mechanism are established and analyzed successively. Firstly, the compliance model of a class of *n*-4R compliant parallel pointing mechanism is established based on the coordinate transformation. The model is verified by finite element analysis, and the influence of geometric parameter variations on the compliance performance of the mechanism is analyzed. Secondly, the mechanism is simplified to an equivalent spring system, and the governing equation of the equivalent spring system is constructed by utilizing the established compliance model. According to the governing equation, the mapping relationship between the input force and the output displacement of the mechanism is subsequently obtained, that is, the kinetostatic model. Then, the accuracy of the kinetostatic model is verified by two simulation examples: The spiral trajectory of the mobile platform center and the spatial pointing trajectory of the mechanism. The results of the two examples show that the deviations between the analytical results and the FE-results are within 0.038% and 0.857%, with the excellent consistency indicating the accuracy of the kinetostatic model. Finally, the influence of the geometric parameter values on the mapping matrix in the kinetostatic model is studied.

## 1. Introduction

In recent years, with the rapid development of precision engineering, the pointing mechanism has been widely used in various fields, such as inter-satellite link [1,2,3], antenna pointing [4,5,6], etc. However, increasing performance requirements for the pointing mechanism make it difficult for traditional series and rigid mechanism to meet the accuracy requirements in the field of micro pointing applications.

The compliant parallel mechanism combines a series of advantages such as high precision, no friction, and no lubrication of the compliant mechanism, and large loading capacity and rapid response of the parallel mechanism [7,8,9], which has triggered scholars to explore the pointing mechanism in the field of compliant parallel mechanisms. Du Z et al. [10] designed a precise compliant parallel pointing mechanism based on the Stewart platform, which can achieve a submicroradian resolution and microradian repeatability. Palpacelli M et al. [11] proposed a redundantly actuated 2-DOF mini pointing device, and analyzed the kinetostatic performance of the device.

In the analysis and design of compliant parallel mechanism, compliance is an important performance indicator, and the compliance model is the basis for the analysis of kinematics, freedom, stiffness, accuracy, and dynamic performance of the mechanism [12]. Lobontiu N. et al. [13] studied the tripod mechanisms that comprise novel spatial Cartesian flexible hinges and can be used in three-dimensional sensing/actuation applications, derived the compliance of mechanism, and analyzed the influence of the geometric parameter on the hinge and tripod analytical compliance. Xiao S. et al. [14] designed a novel compliant flexure-based micro-parallel positioning stage for a micro active vibration isolation application, established the compliance of the mechanism by the compliance matrix method, and the compliance model was verified by FEA-simulation. Zhang D. et al. [15] proposed a six-DOF parallel positioning system with high resolution, high repeatability, and low parasitic motions, established the compliance model of the mechanism based on the matrix method, and conducted experimental research on the working performance of the mechanism.

On the other hand, although the compliant mechanism has the advantages of integral manufacturing, no friction, higher motion accuracy, and is lightweight compared to the rigid mechanism, the kinetostatic of the compliant mechanism (the relationship between input forces and output displacements) cannot be analyzed by kinematics or statics alone as rigid mechanisms are due to the intrinsic coupling between the kinematic and the elastic behavior of the flexure hinge [16], which brings challenges to the kinetostatic modeling of compliant parallel mechanisms with various complex configurations. In the past few decades, relevant scholars have proposed a variety of modeling methods that can be used for the kinetostatic of compliant mechanisms, such as the pseudo rigid body model method, Castigliano’s theorem, elastic beam theory, the compliance matrix method, etc., [17]. Venkiteswaran V. et al. [18] proposed a pseudo-rigid body model with three revolute joints applicable to curved and straight beams, which can define compliant members as models with three revolute joints, making kinematics constraints and statics equations easy to implement. Chen G et al. [19] combined Castigliano’s theorem, the Crotti–Engesser theorem, the beam constraint model, strain energy, and complementary strain energy, and established an energy-based kinetostatic modeling framework for compliant mechanisms. Li Z. et al. [20] designed and analyzed a compliant nanopositioner with dynamically tunable characteristics, and established a kinetostatic model of the nanopositioner by utilizing elastic beam theory and electromagnetic field coupling analysis to predict the variable stiffness property and dynamically tunable characteristics. Li J. et al. [21] established the kinetostatic model of the compliant two-stage differential micro-displacement amplification mechanism by matrix representation and optimized the position and geometric parameters of the flexure hinge. Ling M. et al. [22] proposed a kinetostatic modeling method of compliant mechanisms based on a semi-analytical matrix displacement method, which can be used for complex, compliant mechanisms with serial-parallel substructures. Recently, the authors [23] presented a general approach, which is applicable to describe the kinetostatic and dynamic behaviors of spatial compliant mechanisms. Arredondo-soto M et al. [24] proposed a systematic method for kinetostatic analysis of arbitrary compliant parallel mechanisms based on the compliance matrix method. 

In this paper, a novel class of *n*-4R compliant parallel pointing mechanisms is proposed, and the compliance and kinetostatic of the mechanism are successively modeled and analyzed. Firstly, the compliance of a single branch of the mechanism is derived by using the compliance of right-circular flexure hinges according to coordinate transformation method. Then, the compliance model of the overall mechanism was established according to the flexure module modeling method of the parallel structure. The accuracy of the compliance model was validated by finite element analysis, and the influence of parameter changes on the compliance of the mechanism was further analyzed. Secondly, the mechanism is simplified to an equivalent spring system, and the governing equation of the spring system is established according to Hooke’s law. According to the governing equation, the mapping relationship between input forces and output displacements of this class of *n*-4R compliant parallel pointing mechanisms, that is, the kinetostatic model, is obtained. Finally, the effectiveness of the kinetostatic model was verified by the comparison of analytical calculation and finite element simulation of two trajectories, and the effects of the structure parameter values of the flexure hinge and the number of mechanism branches on the mapping matrix of the kinetostatic model were analyzed.

## 2. Structure of *n*-4R Compliant Parallel Pointing Mechanism

**Figure 1:**
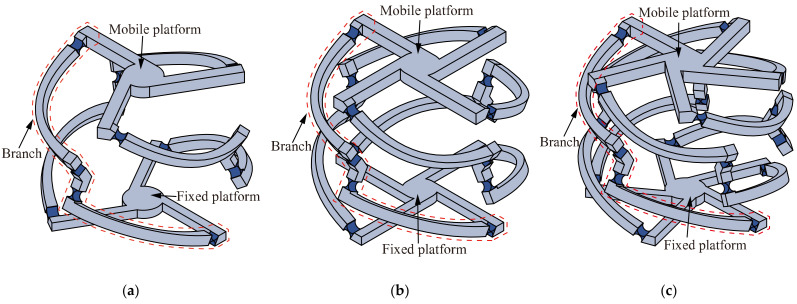
A class of *n*-4R compliant parallel pointing mechanisms, (**a**) *n* = 3; (**b**) *n* = 4; (**c**) *n* = 5.

The *n*-4R compliant parallel pointing mechanism is a class of 2-DOF parallel micro-motion platforms with two rotational DOFs around *x-* and *z*-axes that can realize quasi-sphere motion. As shown in Figure 1, it is composed of a mobile platform, a fixed platform, and *n* (*n* ≥ 3) similar branches. 

**Figure 2:**
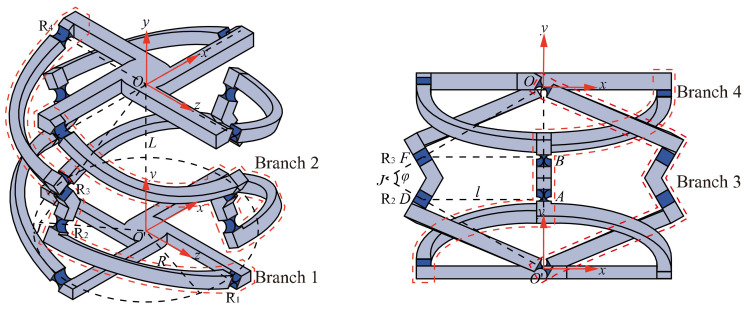
Structure parameters of 4-4R compliant parallel pointing mechanism.

For convenience, the 4-4R compliant parallel pointing mechanism is used as an example to introduce the mechanism structure. As shown in Figure 2, the 4-4R compliant parallel pointing mechanism is composed of a fixed platform, four branches, and a mobile platform. Each branch consists of four right-circular flexure hinges (hereafter referred to as flexure hinges) and three links connected in series. The four branches are similar and equally distributed around the mobile platform by 90°. *O* and *O*′ denote the distribution centers of the flexure hinges directly connected to the mobile and fixed platforms, respectively (hereinafter referred to as the mobile platform center and the fixed platform center, respectively). *O* is defined as the intersection point of the axes of the four flexure hinges connected to the mobile platform, and *O*′ is defined as the intersection point of the axes of the four flexure hinges connected to the fixed platform. We define the distribution radius of both mobile and fixed platforms as *R*, and the distance between the centers of mobile and fixed platforms as *L*. For the flexure hinges *R*_2_ and *R*_3_ on branch 1, their axes pass through *O*′ and *O*, respectively, and intersect at point *J*. From the geometric characteristics, it is known that *OJ* = *O*′*J*. Define the angle ∠*OJO*′ as *φ*. Define the geometric centers of flexure hinges *R*_2_ and *R_3_* as points *D* and *F*, respectively. The horizontal distance from D and F to *OO*′ are both *l*.

## 3. Compliance Model of *n*-4R Compliant Parallel Pointing Mechanism

In this section, firstly, the compliance model of the *n*-4R compliant parallel mechanism is established by the compliance matrix method. Then, the compliance model is verified by Finite Element Analysis. Finally, the influence of the structure parameters of the flexure hinges and the scale parameters of the mechanism on the overall compliance of the mechanism is analyzed.

### 3.1. Compliance of the Right-Circular Flexure Hinge

**Figure 3:**
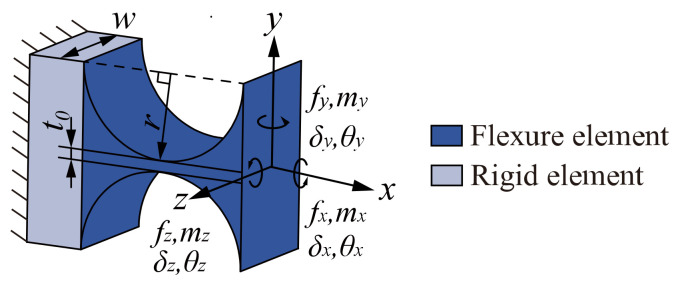
Structure parameters and coordinate frame setting of the right-circular flexure hinge.

Compliance is an important performance indicator of the compliant mechanism, and the compliance of the flexure hinge is the basis for the compliance modeling of the mechanism. Each branch of the *n*-4R compliant parallel pointing mechanism contains four similar right-circular flexure hinges denoted by *R*_1_, *R*_2_, *R*_3_, and *R*_4_. The parameters defining the right-circular flexure hinges are shown in Figure 3, where *r* is the radius of the flexure hinge, *w* is the width of the flexure hinge, and *t*_0_ is the minimum thickness of the flexure hinge along the *x*-axis.

Since the deformation of the right-circular flexure hinge is mainly concentrated in the circular part of the hinge, deformation outside the circular part of the hinge can be ignored. Based on the assumption of small elastic deformation, the deformation of the flexure hinge in all directions should satisfy the principle of linear superposition. Assuming that the force ***f*** = [*f_x_*, *f_y_*, *f_z_*]^T^ and the moment ***m*** = [*m_x_*, *m_y_*, *m_z_*]^T^ act at the end of the flexure hinge, the resulting linear displacement and angular displacement at the end of the hinge are ***δ*** = [*δ_x_*, *δ_y_*, *δ_z_*]^T^ and ***θ*** = [*θ_x_*, *θ_y_*, *θ_z_*]^T^, respectively. Then the relationship between the displacement and the input force at the end of the flexure hinge is defined [25]:

(1) $$\left[\begin{array}{c} \theta_{x}\\ \theta_{y}\\ \theta_{z}\\ \delta_{x}\\ \delta_{y}\\ \delta_{z} \end{array}\right]=\left[\begin{array}{cccccc} C_{\theta_{x},m_{x}} & 0 & 0 & 0 & 0 & 0\\ 0 & C_{\theta_{y},m_{y}} & 0 & 0 & 0 & C_{\theta_{y},f_{z}}\\ 0 & 0 & C_{\theta_{z},m_{z}} & 0 & C_{\theta_{z,}f_{y}} & 0\\ 0 & 0 & 0 & C_{\delta_{x},f_{x}} & 0 & 0\\ 0 & 0 & C_{\delta_{y},m_{z}} & 0 & C_{\delta_{y},f_{y}} & 0\\ 0 & C_{\delta_{z},m_{y}} & 0 & 0 & 0 & C_{\delta_{z},f_{z}} \end{array}\right]\left[\begin{array}{c} m_{x}\\ m_{y}\\ m_{z}\\ f_{x}\\ f_{y}\\ f_{z} \end{array}\right]$$

where the 6 × 6 matrix is compliance matrix ***C*** of the right-circular flexure hinges and is computed in Appendix A.1.

### 3.2. Compliance of the Branch

**Figure 4:**
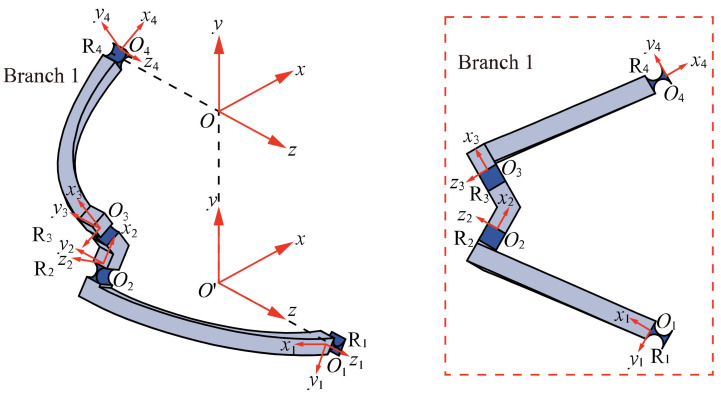
Structure and coordinate frame setting of the branch 1.

As shown in Figure 4, branch 1 consists of four similar flexure hinges denoted by *R*_1_, *R*_2_, *R*_3_, *R*_4_ and three links in series. Compared with the flexure hinge, the stiffness of the link is large enough so that the deformation of the link can be ignored and only the deformation of the flexure hinge is considered.

Coordinate frame setting of the branch 1 is shown in Figure 4. Flexure elements, *R*_1_, *R*_2_, *R*_3_, and *R*_4_, have their own local coordinate frames, *O_1_x_1_y_1_z_1_*, *O_2_x_2_y_2_z_2_*, *O_3_x_3_y_3_z_3_*, and *O_4_x_4_y_4_z_4_*. The global coordinate frame *Oxyz* is fixed at the initial location of the mobile platform center. Since the compliance of the series compliant mechanism is the sum of the compliance of each flexure element in the global coordinate frame [26], the compliance of branch 1 can be then defined as:

(2) $$C_{B1}^{O}=\overset{4}{\underset{i=1}{\sum }}C_{Ri}^{O}$$

where *i* = 1, …, 4 is the number of flexure elements in the branch 1, while $C_{B1}^{O}$ and $C_{Ri}^{O}$ represent the compliance matrix of branch 1 and *R_i_* in the global coordinate frame, respectively.

Assuming that the compliance matrix of flexure hinges *R_i_* in local coordinate frame *O_i_x_i_y_i_z_i_* is $C_{Ri}^{Oi}$, its compliance matrix $C_{Ri}^{O}$ in the global coordinate frame *Oxyz* can be obtained by the following coordinate transformation:

(3) $$C_{Ri}^{O}=Ad_{Oi}^{O}\cdot C_{Ri}^{Oi}\cdot {\left(Ad_{Oi}^{O}\right)}^{T}$$

where *i* = 1, …,4 is the number of flexure elements in the branch 1, and $Ad_{Oi}^{O}$ is the so-called 6 × 6 adjoint transformation matrix, indicating the transformation from local coordinate frame *O_i_x_i_y_i_z_i_* to global coordinate frame *Oxyz.* Assuming that the rotation transformation matrix of the local coordinate frame *O_i_x_i_y_i_z_i_* to the global coordinate frame *Oxyz* is $R_{Oi}^{O}$ and the translation vector is $t_{Oi}^{O}={\left(x,y,z\right)}^{T}$, the adjoint transformation matrix $Ad_{Oi}^{O}$ is then defined as:

(4) $$\begin{array}{c} Ad_{Oi}^{O}=\left[\begin{array}{cc} R_{Oi}^{O} & 0\\ T_{Oi}^{O}\cdot R_{Oi}^{O} & R_{Oi}^{O} \end{array}\right] \end{array},\;\mathrm{where}T_{Oi}^{O}=\left[\begin{array}{ccc} 0 & -z & y\\ z & 0 & -x\\ -y & x & 0 \end{array}\right],\;R_{Oi}^{O}=\left[\begin{array}{ccc} c\beta c\gamma & -c\beta s\gamma & s\beta \\ c\alpha s\gamma +c\gamma s\alpha s\beta & c\alpha c\gamma -s\alpha s\beta s\gamma & -c\beta s\alpha \\ s\alpha s\gamma -c\alpha c\gamma s\beta & c\gamma s\alpha +c\alpha s\beta s\gamma & c\alpha c\beta \end{array}\right]$$

where $T_{Oi}^{O}$ is the antisymmetric matrix defined by the translation vector $t_{Oi}^{O}$. *α*, *β* and *γ* are the angle of rotation around *x-*, *y-,* and *z*-axis, respectively. s and c represent sin and cos, respectively. The parameters for constructing the translation vector $t_{Oi}^{O}$ and rotation matrix $R_{Oi}^{O}$ are listed in Table 1.

Since the structure parameters of all flexure hinges in the *n*-4R compliant parallel pointing mechanism are exactly the same, therefore:

(5) $$C_{R1}^{O1}=C_{R2}^{O2}=C_{R3}^{O3}=C_{R4}^{O4}=C$$

where ***C*** is the compliance matrix of the right-circular flexure hinge, which can be computed in Appendix A.1.

### 3.3. Compliance of the n-4R Compliant Parallel Pointing Mechanism

For the compliant parallel mechanism, the stiffness of the mechanism is the sum of the stiffness of each parallel branch [26], and the compliance matrix and stiffness matrix are mutually inverse matrices. Hence, the compliance of the *n*-4R compliant parallel pointing mechanism is expressed as:

(6) $$C_{n-4R}={\left(\overset{n}{\underset{i=1}{\sum }}{\left(C_{Bi}^{O}\right)}^{-1}\right)}^{-1}$$

where $C_{n-4R}$ is the overall compliance matrix of the *n*-4R compliant parallel pointing mechanism, and $C_{Bi}^{O}$ represents the compliance matrix of branch *i* (*i* = 1, …, *n*) in the global coordinate frame.

Since the branches of the *n*-4R compliant parallel pointing mechanism are similar and evenly distributed, the compliance matrix $C_{B2}^{O}$ of branch 2 can be obtained by rotating the compliance matrix $C_{B1}^{O}$ of branch 1 by 2π/n angle around the *y*-axis of the global coordinate frame *Oxyz*.

(7) $$C_{Bi}^{O}=Ad_{R}C_{B(i-1)}^{O}Ad_{R}^{T},\;\mathrm{where}Ad_{R}=\left[\begin{array}{cc} R_{y,2\pi /n} & 0\\ 0 & R_{y,2\pi /n} \end{array}\right]$$

where $Ad_{R}$ is the adjoint transformation matrix, $R_{y,2\pi /n}$ is the rotation matrix, which represent the rotation of 2π/n angle around the *y*-axis of the global coordinate frame *Oxyz*, *n* (*n* ≥ 3) is the number of branches, and *i* represents the *i*-th branch, *i* = 2, …,*n*.

### 3.4. Validation of Compliance Model with Computational Simulations 

In this section, the 4-4R compliant parallel pointing mechanism is taken as an example for the verification of the compliance model by FEA simulation assuming small displacements in the commercial software ANSYS2019^®^. The structure parameters of the 4-4R compliant parallel pointing mechanism are listed in Table 2. Young’s modulus of the material of the flexure hinge was set as *E* = 206 GPa and Poisson’s ratio was set as *ν* = 0.3.

By substituting the structural parameters into Equation (6), the overall compliance matrix of the 4-4R compliant parallel pointing mechanism was obtained:

$$C_{4-4R}^{\mathrm{An}}=[\begin{array}{cccccc} C_{\theta_{x},m_{x}} & C_{\theta_{x},m_{y}} & C_{\theta_{x},m_{z}} & C_{\theta_{x},f_{x}} & C_{\theta_{x}f_{y}} & C_{\theta_{x},f_{z}}\\ C_{\theta_{y},m_{x}} & C_{\theta_{y},m_{y}} & C_{\theta_{y},m_{z}} & C_{\theta_{y},f_{x}} & C_{\theta_{y}f_{y}} & C_{\theta_{y},f_{z}}\\ C_{\theta_{z},m_{x}} & C_{\theta_{z},m_{y}} & C_{\theta_{z},m_{z}} & C_{\theta_{z},f_{x}} & C_{\theta_{z,}f_{y}} & C_{\theta_{z},f_{z}}\\ C_{\delta_{x},m_{x}} & C_{\delta_{x},m_{y}} & C_{\delta_{x},m_{z}} & C_{\delta_{x},f_{x}} & C_{\delta_{x}f_{y}} & C_{\delta_{x},f_{z}}\\ C_{\delta_{y},m_{x}} & C_{\delta_{y},m_{y}} & C_{\delta_{y},m_{z}} & C_{\delta_{y},f_{x}} & C_{\delta_{y},f_{y}} & C_{\delta_{y},f_{z}}\\ C_{\delta_{z},m_{x}} & C_{\delta_{z},m_{y}} & C_{\delta_{z},m_{z}} & C_{\delta_{z},f_{x}} & C_{\delta_{z}f_{y}} & C_{\delta_{z},f_{z}} \end{array}]=\left[\begin{array}{cccccc} 7.31\times 10^{-2} & 0 & 0 & 1.43\times 10^{-5} & 0 & 3.65\times 10^{-3}\\ 0 & 3.00\times 10^{-3} & 0 & 0 & 9.15\times 10^{-5} & 0\\ 0 & 0 & 7.30\times 10^{-2} & -3.65\times 10^{-3} & 0 & 1.43\times 10^{-5}\\ 1.43\times 10^{-5} & 0 & -3.65\times 10^{-3} & 2.12\times 10^{-4} & 0 & 0\\ 0 & 9.15\times 10^{-5} & 0 & 0 & 5.28\times 10^{-5} & 0\\ 3.65\times 10^{-3} & 0 & 1.43\times 10^{-5} & 0 & 0 & 2.12\times 10^{-4} \end{array}\right]$$

The element in row *i* and column *j* of the compliance matrix represents the displacement of the mobile platform center in the *i*-direction under the action of the unit load in the *j*-direction.

The validation of analytical results in the compliance model of the 4-4R compliant parallel pointing mechanism is provided by commercial software ANSYS 19.2. A tetrahedron mesh with an element size of 2 mm was created for the links, mobile platform, and fixed platform, and mesh refinements of 0.3 mm were performed at the right-circular flexure hinges. First, we fix the fixed platform of the mechanism, and apply the unit load to the mobile platform center. Therefore, the displacement of the mobile platform center is the compliance of the mechanism in this direction. We repeat this process to obtain the compliance matrix of the mechanism. The compliance matrix obtained by the finite element method is as follows:

$$C_{4-4R}^{\mathrm{FE}}=\left[\begin{array}{cccccc} 7.24\times 10^{-2} & 0 & 0 & 1.52\times 10^{-5} & 0 & 3.62\times 10^{-3}\\ 0 & 3.20\times 10^{-3} & 0 & 0 & 9.86\times 10^{-5} & 0\\ 0 & 0 & 7.24\times 10^{-2} & -3.62\times 10^{-3} & 0 & 1.52\times 10^{-5}\\ 1.52\times 10^{-5} & 0 & -3.62\times 10^{-3} & 2.12\times 10^{-4} & 0 & 0\\ 0 & 9.86\times 10^{-5} & 0 & 0 & 5.50\times 10^{-5} & 0\\ 3.62\times 10^{-3} & 0 & 1.52\times 10^{-5} & 0 & 0 & 2.12\times 10^{-4} \end{array}\right]$$

The comparison of analytical results and the FE-results of compliance is listed in Table 3. It can be seen from Table 3 that the compliance of the main functional direction (rotation direction around the *x*-axis and *z*-axis) in the compliance matrix is the same and much larger than those of the nonfunctional direction (rotation direction around the *y*-axis), indicating that the design of the mechanism and the selection of structure parameters of the flexure hinge are reasonable. In the compliance matrix, the relative errors of the six compliance on the main diagonal are less than 7%, and the relative errors of the functional direction are less than 1%. The error is within the allowable range, which verifies the correctness of the compliance model. The main reasons for the errors are (1) the theoretical compliance model of flexure hinge, and (2) during the theoretical modeling of the overall compliance of the mechanism, the links, mobile platform, and fixed platform are considered rigid elements without deformation, while they are treated as flexure elements in ANSYS, which will deform slightly even if the stiffness is large enough.

**Figure A1:**
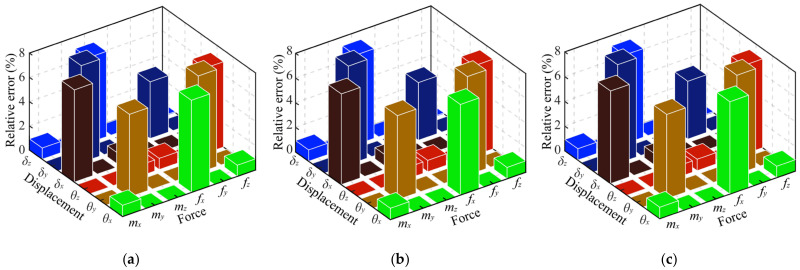
Relative error in percentage between analytical and FE results during the compliance model: (**a**) 3-4R; (**b**) 4-4R; (**c**) 5-4R.

The analytical results, FE-results of the overall compliance matrix of 3-4R and 5-4R compliant parallel pointing mechanisms are given in Appendix A.2, and relative errors of analytical results are shown in Figure A1.

### 3.5. Analysis of Compliance Performance of n-4R Compliant Parallel Pointing Mechanisms

According to the compliance model established in Section 3.3, the structure parameters of the flexure hinge, the scale parameters of the mechanism, and the number of mechanism branches will affect the compliance of the mechanism. Therefore, it is necessary to analyze the influence of the changes of these parameters on the overall compliance of the mechanism. For convenience, ***C****_θ_* and ***C****_δ_* are defined as the rotation-related and translation-related compliance on the diagonal of the compliance matrix, respectively.

**Figure 5:**
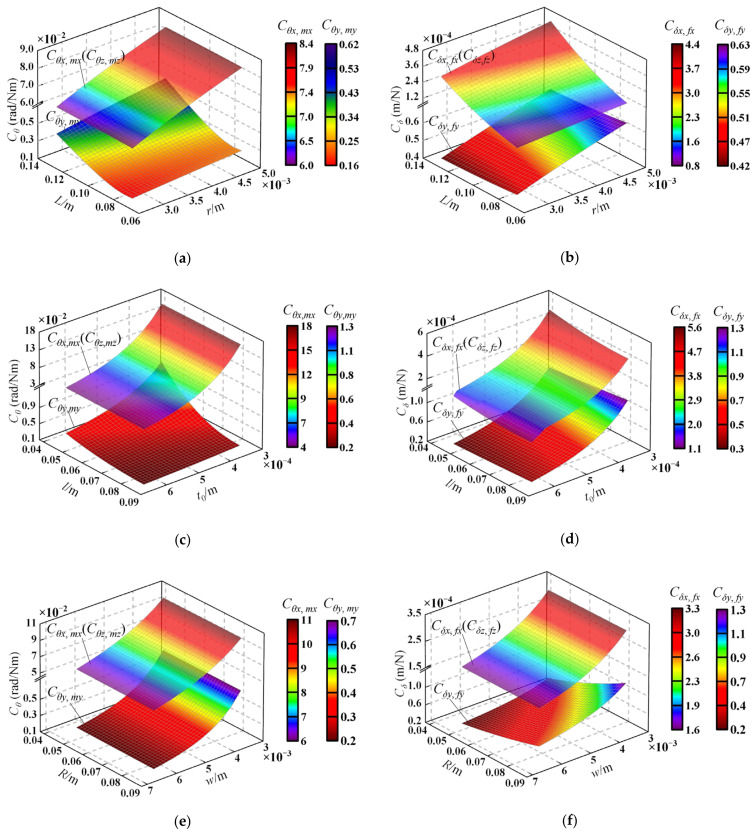
Compliance variation with the structure parameters of the flexure hinge and the scale parameters of the mechanism: (**a**) variation of ***C****_θ_* in terms of the parameters *L* and *r*; (**b**) variation of ***C****_δ_* in terms of the parameters *L* and *r*; (**c**) variation of ***C****_θ_* in terms of the parameters *l* and *t*_0_; (**d**) variation of ***C****_δ_* in terms of the parameters *l* and *t*_0_; (**e**) variation of ***C****_θ_* in terms of the parameters *R* and *w*; (**f**) variation of ***C****_δ_* in terms of the parameters *R* and *w*.

We take the 4-4R compliant parallel pointing mechanism as an example to analyze the influence of structure parameters on the compliance of the mechanism. The analysis is carried out from the following three aspects: (1) The influence of the radius *r* of flexure hinge and the distance *L* between the mobile and fixed platform on ***C****_θ_* and ***C****_δ_*; (2) the influence of parameter *l* and the minimum thickness *t*_0_ of flexure hinge on ***C****_θ_* and ***C****_δ_*; (3) the influence of the distribution radius *R* of mobile and fixed platforms and the width *w* of flexure hinge on ***C****_θ_* and ***C****_δ_*. When analyzing the effect of parameter changes on compliance performance, the parameters in Table 4 were selected, while the other parameters remained the same as in Table 2. Figure 5 shows the variations of diagonal elements of the compliance matrix in terms of the compliance hinge structure parameters and the mechanism scale parameters.

The following conclusions can be drawn from Figure 5: (1)***C**_θ_* and ***C****_δ_* are directly correlated with *L* and *r*, while they are inversely correlated with *l*, *t*_0_, *R,* and *w*. (2) Compared with the structural parameters (*L*, *l*, and *R*) of the mechanism, ***C****_θ_* and ***C****_δ_* are more sensitive to the flexure hinge structure parameters (*r*, *t*_0_, and *w*). Therefore, when designing the mechanism, the structure parameters of the flexure hinge can be changed preferentially to ensure the mechanism meets the compliance performance requirements. (3) The compliance of the mechanism in the functional direction $C_{\theta_{x},m_{x}}$ and $C_{\theta_{z},m_{z}}$ are equal and far greater than those in the non-functional direction $C_{\theta_{y},m_{y}}$, which is consistent with the conclusion in Section 3.4.

**Figure 6:**
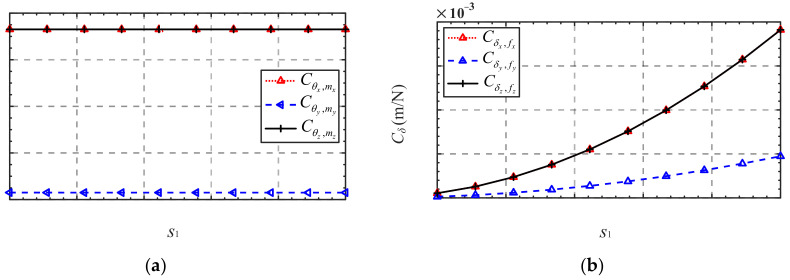
Compliance variation in terms of the scale coefficient *s*_1_: (**a**) ***C**_θ_*; (**b**) ***C**_δ_*.

**Figure 7:**
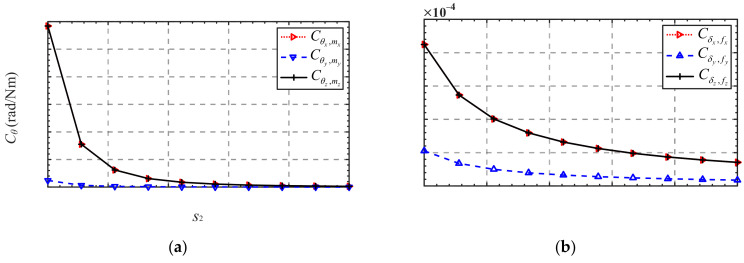
Compliance variation in terms of the scale coefficient *s*_2_: (**a**) ***C**_θ_*; (**b**) ***C**_δ_*.

The influence of parameter variations near a given flexure hinge structure parameter and mechanism scale parameter on the compliance of the mechanism was investigated. However, in practical applications, the scale of the mechanism required for different application scenarios often varies greatly and may differ by several times or even tens of times. Therefore, it is also of interest to qualify and quantify the mechanism compliance scales with the defining geometric dimensions. Considering two different scaling situations, we define the scale coefficients s_1_ and *s*_2_. The scale coefficient *s*_1_ denotes that only dimensional parameters of the mechanism are scaled by *s*_1_ times, while the structural parameters of the flexure hinge remain unchanged. The scale coefficient *s*_2_ denotes that both dimensional parameters of the mechanism and the structural parameters of the flexure hinge are scaled by *s*_2_ times. The analysis results are given in Figure 6 and Figure 7.

As shown in Figure 6 and Figure 7, ***C****_θ_* will not be affected when the scale coefficient *s*_1_ increases, and ***C****_δ_* is directly correlated with the scale coefficient *s*_1_, while ***C****_θ_* and ***C****_δ_* decrease rapidly with the increase in the scale coefficient *s*_2_. Furthermore, ***C****_θ_* is more affected by the scale factor *s*_2_ compared with ***C****_δ_*. The reason for this is that ***C****_θ_* is mainly determined by the compliance of the functional direction of the flexure hinge, which is more easily affected than that of the nonfunctional direction when the flexure hinge is scaled in equal proportion. 

**Figure 8:**
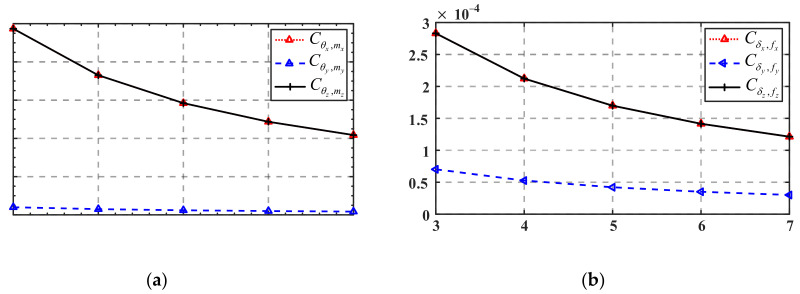
Compliance variation in terms of the number of mechanism branches *n*: (**a**) ***C**_θ_*; (**b**) ***C**_δ_*.

Finally, the influence of the number of mechanism branches *n* on the overall compliance of the *n*-4R compliant parallel pointing mechanism is analyzed. As shown in Figure 8, ***C****_θ_* and ***C****_δ_* decrease when the number of branches *n* increases from 3 to 7, i.e., the overall stiffness of the mechanism increases. This provides an idea for the compliance design of the mechanism without changing the dimension parameters of the mechanism and the structural parameters of the flexure hinge.

## 4. Kinetostatic Model of *n*-4R Compliant Parallel Pointing Mechanism

In Section 3, the compliance model of the *n*-4R compliant parallel pointing mechanism is established. According to the compliant model, the relationship between force and displacement at the mobile platform center at the end of the mechanism is obtained. However, for the compliant mechanism, the input force and the output displacement are usually not in the same coordinate frame, for example, the displacement at the mobile platform center is solved by acting a load on the mechanism branch. In this section, therefore, the kinetostatic model of the *n*-4R compliant parallel pointing mechanism will be analyzed to investigate the mapping relationship between the input force and output displacement in different coordinate frames during slow loading (neglecting the inertial force).

**Figure 9:**
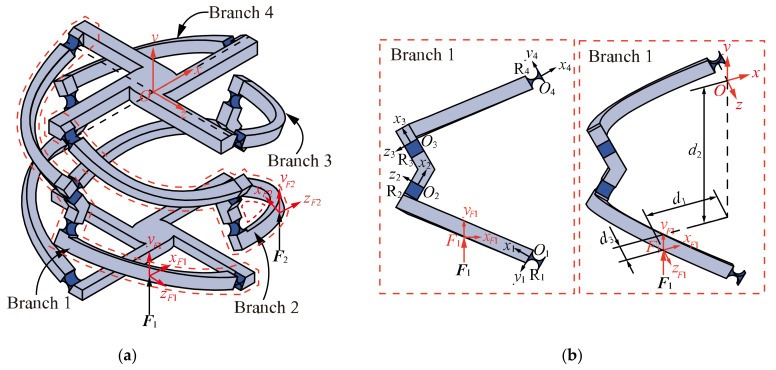
(**a**) Complete overview of the 4-4R compliant parallel pointing mechanism to analyze; (**b**) coordinate frame setting and loading position of force ***F***_1_.

We take the 4-4R compliant parallel pointing mechanism as an example to analyze the kinetostatic model. Since the mechanism has two degrees of freedom, two drives are required for the mechanism to have definite motion. As shown in Figure 9, input forces ***F***_1_ = ${\left[m_{1,x},m_{1,y},m_{1,z},f_{1,x},f_{1,y},f_{1,z}\right]}^{T}$ relative to the local coordinate frame *F*_1_*x_F_*_1_*y_F_*_1_*z_F_*_1_, ***F***_2_ = ${\left[m_{2,x},m_{2,y},m_{2,z},f_{2,x},f_{2,y},f_{2,z}\right]}^{T}$ relative to the local coordinate frame *F*_2_*x_F_*_2_*y_F_*_2_*z_F_*_2_, respectively, on branch 1 and branch 2 and then the mobile platform center generate a certain displacement ***U***_4-4R_ = ${\left[\theta_{x},\theta_{y},\theta_{z},\delta_{x},\delta_{y},\delta_{z}\right]}^{T}$ relative to the global coordinate frame *Oxyz*. If the deformations are in the linear range, the displacements ***U***_1_ and ***U***_2_ can be analyzed under the action of forces ***F***_1_ and ***F***_2_ separately. Then, the relationship between the displacement ***U***_4-4R_ and the forces ***F***_1_ and ***F***_2_ can be analyzed by using the principle of superposition.

### 4.1. Relationship between Input Force F_1_ and Output Displacement U_1_

**Figure 10:**
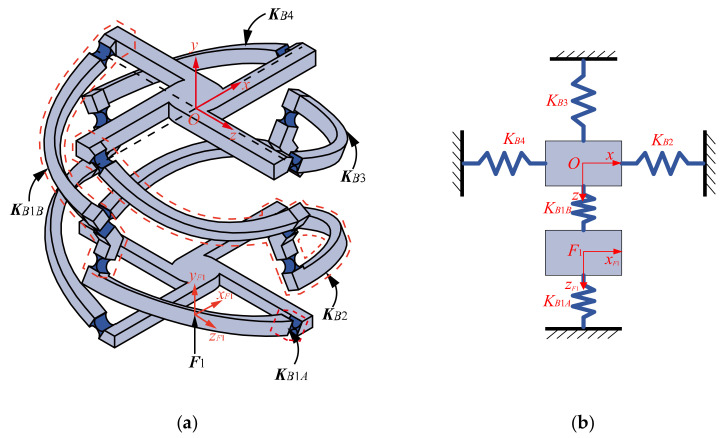
4-4R compliant parallel pointing mechanism subjected to force ***F***_1_: (**a**) Simplification of equivalent stiffness; (**b**) equivalent spring system.

For the convenience of analysis, the mechanism is simplified as an equivalent spring system, and the concept of equivalent stiffness is introduced [24,27]. As shown in Figure 10, ***K****_B_*_1*A*_ is defined as the equivalent stiffness matrix of the part between the loading position on branch 1 and the fixed platform. ***K****_B_*_1*B*_ is defined as the equivalent stiffness matrix of the part between the loading position on branch 1 and the mobile platform. ***K****_B_*_2_, ***K****_B_*_3_, and ***K****_B_*_4_ are the equivalent stiffness matrices of branch 2, 3, and 4, respectively. Therefore, according to Hooke’s law, the governing equations of the elastic deformation of the system is expressed as:

(8) $$\left[\begin{array}{cc} {\left(K_{OO}\right)}_{F_{1}} & K_{OF_{1}}\\ K_{F_{1}O} & K_{F_{1}F_{1}} \end{array}\right]\left[\begin{array}{c} U_{1}\\ U_{F_{1}} \end{array}\right]=\left[\begin{array}{c} F_{O}\\ F_{1} \end{array}\right]$$

where ***U***_1_ represents the displacement of the mobile platform center relative to the global coordinate frame *Oxyz*, ***U****_F_*_1_ represents the displacement of the action point of force ***F***_1_ relative to coordinate frame *F*_1_*x_F_*_1_*y_F_*_1_*z_F_*_1_, and ***F****_O_* represents the force acting on the mobile platform center.

The stiffness matrices in the governing equations of spring system are computed by Equation (9).

(9) $$\left\{\begin{array}{l} {\left(K_{OO}\right)}_{F_{1}}=K_{B1B}^{O}+K_{B2}^{O}+K_{B3}^{O}+K_{B4}^{O}\\ \begin{array}{c} K_{F_{1}F_{1}}=K_{B1A}^{F_{1}}+K_{B1B}^{F_{1}} \end{array}\\ \begin{array}{c} K_{OF_{1}}=-{\left(Ad_{O}^{O}\right)}^{-T}\left[K_{B1B}^{O}\right]{\left(Ad_{O}^{F_{1}}\right)}^{-1} \end{array}\\ \begin{array}{c} K_{F_{1}O}=-{\left(Ad_{O}^{F_{1}}\right)}^{-T}\left[K_{B1B}^{O}\right]{\left(Ad_{O}^{O}\right)}^{-1} \end{array} \end{array}\right.$$

where the superscripts *O* and *F*_1_ of each stiffness matrix indicate that the stiffness matrix is relative to coordinate frame *Oxyz* and *F*_1_*x_F_*_1_*y_F_*_1_*z_F_*_1_, respectively.

$$Ad_{O}^{O}=I_{6\times 6},Ad_{O}^{F_{1}}=\left[\begin{array}{cc} R_{O}^{F_{1}} & 0\\ T_{O}^{F_{1}}\cdot R_{O}^{F_{1}} & R_{O}^{F_{1}} \end{array}\right],\;\mathrm{where}\;T_{O}^{F_{1}}=\left[\begin{array}{ccc} 0 & d_{3} & d_{2}\\ -d_{3} & 0 & -d_{1}\\ -d_{2} & d_{1} & 0 \end{array}\right],\;R_{O}^{F_{1}}=I_{3\times 3}$$

where ***I*** is the unit matrix. *D*_1_, *d*_2_, and *d*_3_ represent loading position of force ***F***_1_ relative to global coordinate frame *Oxyz*.

The stiffness matrices in Equation (9) can be calculated from Equations (2), (3), and (7):

(10) $$\left\{\begin{array}{l} K_{B1A}^{O}={\left(C_{R1}^{O}\right)}^{-1},K_{B1B}^{O}={\left(C_{R2}^{O}+C_{R3}^{O}+C_{R4}^{O}\right)}^{-1}\\ K_{B2}^{O}={\left(C_{B2}^{O}\right)}^{-1},{K_{B3}^{O}=\left(C_{B3}^{O}\right)}^{-1},{K_{B4}^{O}=\left(C_{B4}^{O}\right)}^{-1}\\ K_{B1A}^{F_{1}}={\left({\mathrm{Ad}}_{O}^{F_{1}}{\left(K_{B1A}^{O}\right)}^{-1}{\left({\mathrm{Ad}}_{O}^{F_{1}}\right)}^{T}\right)}^{-1}\\ K_{B1B}^{F_{1}}={\left({\mathrm{Ad}}_{O}^{F_{1}}{\left(K_{B1B}^{O}\right)}^{-1}{\left({\mathrm{Ad}}_{O}^{F_{1}}\right)}^{T}\right)}^{-1} \end{array}\right.$$

Since there is no force applied to the mobile platform, ***F**_O_* in Equation (8) can be set to be ***0***, which yields:

(11) $$\begin{array}{c} U_{1}=C_{TOF_{1}}F_{1} \end{array}$$

where:

(12) $$\begin{array}{c} \;C_{TOF_{1}}=-{\left({\left(K_{OO}\right)}_{F_{1}}-K_{OF_{1}}K_{F_{1}F_{1}}^{-1}K_{F_{1}O}\right)}^{-1}\left(K_{OF_{1}}K_{F_{1}F_{1}}^{-1}\right) \end{array}$$

So far, the relationship between the displacement ***U***_1_ of the mobile platform center relative to the coordinate frame *Oxyz* and the force ***F***_1_ relative to the coordinate frame *F*_1_*x_F_*_1_*y_F_*_1_*z_F_*_1_ can be described by mapping matrix $C_{TOF_{1}}$.

### 4.2. Kinetostatic Model of 4-4R Compliant Parallel Pointing Mechanism

When the forces ***F***_1_ and ***F***_2_ act on the mobile platform simultaneously, displacement ***U***_4-4R_ at the center of the mobile platform can be regarded as the superposition of the displacement ***U***_1_ and ***U***_2_ at the center of the mobile platform under the separate action of force ***F***_1_ and ***F***_2_. Therefore, the total displacement of mobile platform is defined as:

(13) $$U_{4-4R}=U_{1}+U_{2}$$

According to Equations (11) and (13), one can obtain:

(14) $$\begin{array}{c} U_{4-4R}=\left[C_{TOF_{1}}\;\;C_{TOF_{2}}\right]\left[\begin{array}{c} F_{1}\\ F_{2} \end{array}\right] \end{array}$$

where $C_{TOF_{2}}$ is the mapping matrix between force ***F***_2_ and displacement ***U***_2_ of mobile platform center.

Due to the symmetry of the structure, the mapping relationship between the force ***F***_2_ and the displacement ***U***_2_ of the mobile platform center can be obtained by rotation transformation of relative elements in Equation (8). We define a new adjoint transformation matrix as:

(15) $$\begin{array}{c} Ad_{\pi /2}=\left[\begin{array}{cc} R_{y,\pi /2} & 0\\ 0 & R_{y,\pi /2} \end{array}\right] \end{array}$$

where $R_{y,\pi /2}$ is the rotation matrix, representing 90° rotation around the *y*-axis of global coordinate frame *Oxyz*.

The coordinate transformation of related elements in Equation (8) yields:

(16) $$\left\{\begin{array}{l} {\left(K_{OO}\right)}_{F_{2}}=Ad_{\pi /2}\left[{\left(K_{OO}\right)}_{F_{1}}\right]{\left(Ad_{\pi /2}\right)}^{T}\\ K_{F_{2}F_{2}}=K_{F_{1}F_{1}}\\ K_{OF_{2}}=\mathrm{AA}d_{\pi /2}\left[K_{OF_{1}}\right]\\ K_{F_{2}O}=\left[K_{F_{1}O}\right]{\left(Ad_{\pi /2}\right)}^{T} \end{array}\right.$$

By rearranging Equations (12) and (16), one can obtain:

$$C_{TOF_{2}}={\left[Ad_{\pi /2}\right]}^{-T}\left[-{\left({\left(K_{OO}\right)}_{F_{1}}-K_{OF_{1}}K_{F_{1}F_{1}}^{-1}K_{F_{1}O}\right)}^{-1}\left(K_{OF_{1}}K_{F_{1}F_{1}}^{-1}\right)\right]$$

The above equation can be further simplified as:

(17) $$\begin{array}{c} C_{TOF_{2}}={\left[Ad_{\pi /2}\right]}^{-T}\left[C_{TOF_{1}}\right] \end{array}$$

So far, the kinetostatic model of the 4-4R compliant parallel pointing mechanism has been established. 

### 4.3. Kinetostatic Model of n-4R Compliant Parallel Pointing Mechanism

In this section, the kinetostatic model is extended from a 4-4R to a class of *n*-4R compliant parallel pointing mechanism. It can be seen from Equation (9) that only the parameters $K_{B2}^{O}$, $K_{B3}^{O}$, and $K_{B4}^{O}$ are associated with the number of mechanism’s branches, and Equation (9) can thus be rewritten as follows:

(18) $$\left\{\begin{array}{l} {\left(K_{OO}\right)}_{F_{1}}=K_{B1B}^{O}+K_{4-4R}-K_{B1}^{O}\\ K_{F_{1}F_{1}}=K_{B1B}^{F_{1}}+K_{B1A}^{F_{1}}\\ K_{OF_{1}}=-{\left(A,d_{O}^{O}\right)}^{-T}\left[K_{B1B}^{O}\right]{\left(Ad_{O}^{F_{1}}\right)}^{-1}\\ K_{F_{1}O}=-{\left(Ad_{O}^{F_{1}}\right)}^{-T}\left[K_{B1B}^{O}\right]{\left(Ad_{O}^{O}\right)}^{-1} \end{array}\right.$$

where ***K***_4-4R_ denotes the overall stiffness of the 4-4R compliant parallel pointing mechanism, and $K_{B1}^{O}$ denotes the stiffness of branch 1.

According to Equation (18), it is easy to derive the general equation of the mapping matrix $C_{TOF_{1}}$ between the force ***F***_1_ applied to the branch 1 and the displacement ***U***_1_ of the mobile platform center in the kinetostatic model of the *n*-4R compliant parallel pointing mechanism.

(19) $$\begin{array}{c} \;C_{TOF_{1}}=-{\left({\left(K_{OO}\right)}_{F_{1}}-K_{OF_{1}}K_{F_{1}F_{1}}^{-1}K_{F_{1}O}\right)}^{-1}\left(K_{OF_{1}}K_{F_{1}F_{1}}^{-1}\right) \end{array}$$

where:

(20) $$\left\{\begin{array}{l} {\left(K_{OO}\right)}_{F_{1}}=K_{B1B}^{O}+K_{n-4R}-K_{B1}^{O}\\ K_{F_{1}F_{1}}=K_{B1B}^{F_{1}}+K_{B1A}^{F_{1}}\\ K_{OF_{1}}=-{\left(Ad_{O}^{O}\right)}^{-T}\left[K_{B1B}^{O}\right]{\left(Ad_{O}^{F_{1}}\right)}^{-1}\\ K_{F_{1}O}=-{\left(Ad_{O}^{F_{1}}\right)}^{-T}\left[K_{B1B}^{O}\right]{\left(Ad_{O}^{O}\right)}^{-1} \end{array}\right.$$

where:

(21) $$\left\{\begin{array}{l} K_{B1A}^{O}={\left(C_{R1}^{O}\right)}^{-1},K_{B1B}^{O}={\left(C_{R2}^{O}+C_{R3}^{O}+C_{R4}^{O}\right)}^{-1}\\ K_{B1}^{O}={\left(C_{B1}^{O}\right)}^{-1},K_{n-4R}={\left(C_{n-4R}\right)}^{-1}\\ K_{B1A}^{F_{1}}={\left(Ad_{O}^{F_{1}}{\left(K_{B1A}^{O}\right)}^{-1}{\left(Ad_{O}^{F_{1}}\right)}^{T}\right)}^{-1}\\ K_{B1B}^{F_{1}}={\left(Ad_{O}^{F_{1}}{\left(K_{B1B}^{O}\right)}^{-1}{\left(Ad_{O}^{F_{1}}\right)}^{T}\right)}^{-1} \end{array}\right.$$

where $K_{B1}^{O}$ denotes the stiffness of the branch 1 in the coordinate frame *Oxyz* and ***K****_n_*_-4R_ denotes the overall compliance matrix of the *n*-4R mechanism.

Similar to Equation (17), the mapping relationship $C_{TOF_{2}}$ between the force ***F***_2_ applied to branch 2 and the displacement ***U***_2_ of the mobile platform center can be simplified to a rotational transformation of $C_{TOF_{1}}$, and the angle of rotation is determined by the number of the mechanism’s branches.

(22) $$\begin{array}{c} C_{TOF_{2}}={\left[Ad_{R}\right]}^{-T}\left[C_{TOF_{1}}\right] \end{array},\;\mathrm{where}Ad_{R}=\left[\begin{array}{cc} R_{y,2\pi /n} & 0\\ 0 & R_{y,2\pi /n} \end{array}\right]$$

where $R_{y,2\pi /n}$ is the rotation matrix, indicating a rotation of 2π/n angle around the *y*-axis of the global coordinate frame *Oxyz*.

According to the principle of superposition, the relationship between the displacement of the mobile platform center and the forces ***F***_1_ and ***F***_2_ can be obtained as follows:

(23) $$\begin{array}{c} U_{n-4R}=\left[C_{TOF_{1}}\;\;C_{TOF_{2}}\right]\left[\begin{array}{c} F_{1}\\ F_{2} \end{array}\right] \end{array}$$

where ***U****_n_*_-4R_ denotes the displacement of the mobile platform center of the *n*-4R mechanism by applying forces ***F***_1_ and ***F***_2_ simultaneously.

## 5. Validation and Analysis of Kinetostatic Model with Computational Simulations

This section takes the 4-4R mechanism as an example to verify the accuracy of the kinetostatic model by comparing the theoretical calculation and finite element simulation of the two examples. Thereafter, the influence of the structure parameters of the flexure hinge and the number of mechanism’s branches on the mapping relationship between the input force and output displacement in different coordinate frames is analyzed.

### 5.1. Computational Simulation of Spiral Trajectory

**Figure 11:**
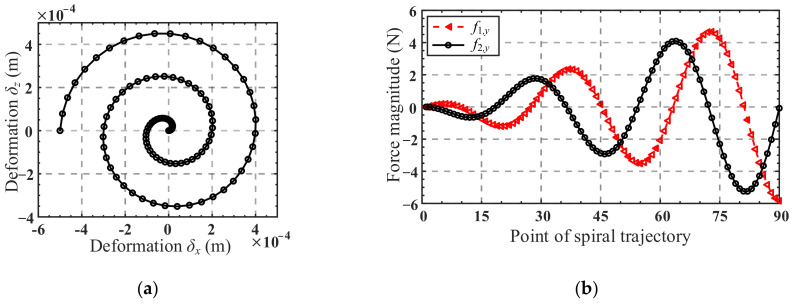
(**a**) Analytical spiral trajectory; (**b**) curves of the input forces *f*_1,*y*_, *f*_2,*y*_.

As shown in Figure 11a, let the center of the mobile platform move along a given spiral trajectory in the *x*-direction and *z*-direction, and the equation of the trajectory is as follows:

(24) $$\left\{\begin{array}{c} \delta_{x}=R_{c}\cos(10000R_{c}\pi )\\ \delta_{z}=R_{c}\sin(10000R_{c}\pi ) \end{array}\right.,\;0\leq R_{c}\leq 5\times 10^{-4}\;m$$

where *δ_x_* and *δ_z_* denote the displacement of the mobile platform center in the *x*-direction and *z*-direction, respectively, and *R*_c_ is the radius of the spiral trajectory.

Suppose the forces *f*_1,*y*_ and *f*_2,*y*_ along the *y*-direction are applied at loading position of forces ***F***_1_ and ***F***_2_ on branch 1 and branch 2, respectively. According to Equation (14), the mapping relationship between force *f*_1,*y*_, *f*_2,*y*_ and the displacement *δ_x_*, *δ_z_* of the mobile platform center can be obtained:

(25) $$\left[\begin{array}{c} \delta_{x}\\ \delta_{z} \end{array}\right]=\left[\begin{array}{c} \begin{array}{cc} C_{\delta_{x},f_{1,y}} & C_{\delta_{x},f_{2,y}}\\ C_{\delta_{z},f_{1,y}} & C_{\delta_{z},f_{2y}} \end{array} \end{array}\right]\left[\begin{array}{c} f_{1,y}\\ f_{2,y} \end{array}\right]=\left[\begin{array}{c} \begin{array}{cc} {\left(C_{TOF_{1}}\right)}_{\left[rows4,6,col5\right]} & {\left(C_{TOF_{2}}\right)}_{\left[rows4,6,col5\right]} \end{array} \end{array}\right]\left[\begin{array}{c} f_{1,y}\\ f_{2,y} \end{array}\right]$$

where ${\left(C_{TOF_{1}}\right)}_{\left[rows4,6,col5\right]}$ denotes the elements of the fifth column of the fourth and sixth rows of the mapping matrix $C_{TOF_{1}}$, and ${\left(C_{TOF_{2}}\right)}_{\left[rows4,6,col5\right]}$ denotes the elements of the fifth column of the fourth and sixth rows of the mapping matrix $C_{TOF_{2}}$. We define the matrix consisting of $C_{\delta_{x},f_{1,y}}$,$C_{\delta_{x},f_{2,y}}$,$C_{\delta_{z},f_{1,y}}$ and $C_{\delta_{z},f_{2y}}$ as the translation mapping matrix.

**Figure 12:**
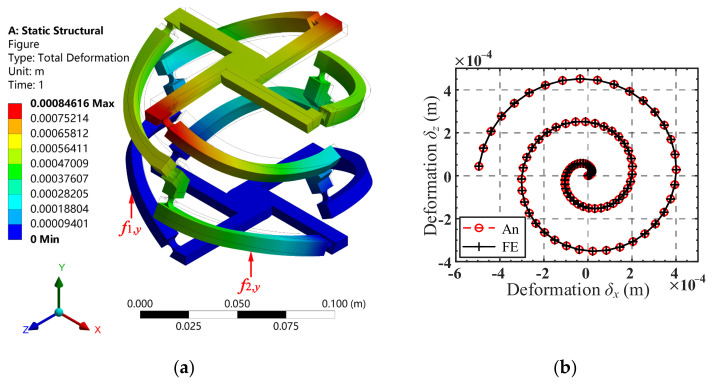
(**a**) Total-deformation results plot of the FE-model under the set of forces; (**b**) analytical and FE spiral trajectories.

A total of 90 points, obtained by equally spacing the domain 0 < *R*_c_ < 5 × 10^−4^ m, were used to form the spiral trajectory. The displacements *δ_x_* and *δ_z_* of the 90 points were substituted in Equation (25) successively to obtain the corresponding input forces *f*_1,*y*_ and *f*_2,*y*_. In the process, the structure parameters of the mechanism and the loading position of force ***F***_1_ are listed in Table 2, and the loading position of force ***F***_2_ can be obtained from the loading position of force ***F***_1_ by rotation transformation. Curves of the input forces are shown in Figure 11b. Using the input forces for Finite Element Analysis (a computational model of the 4-4R mechanism was constructed with the dimensions listed in Table 2). Young’s modulus of the material of the flexure hinge was set as *E* = 206 GPa and Poisson’s ratio was set as *ν* = 0.3. A tetrahedron mesh with an element size of 2 mm was created for this model, and mesh refinements of 0.3 mm were performed at the flexure hinge. The corresponding trajectory of the center of the mobile platform can thus be obtained, as shown in Figure 12a.

**Figure 13:**
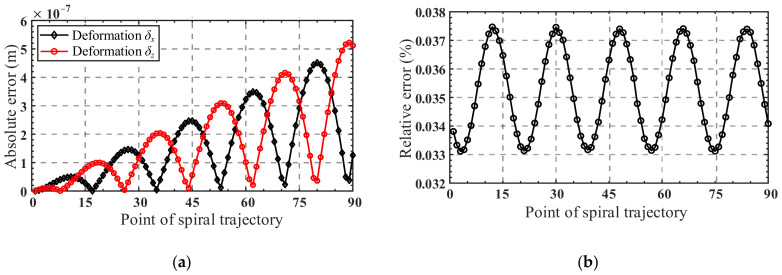
(**a**) Absolute errors of displacements *δ_x_* and *δ_z_*, during spiral trajectory; (**b**) relative errors in percentage, between analytical and FE-results for both trajectories during the spiral trajectory.

A comparison of the analytical and FE-result of spiral trajectory is shown in Figure 12b. As can be seen from Figure 12b, the analytical spiral trajectory agrees well with the simulated one, indicating the correctness of the kinetostatic model. The absolute error and relative error of the analytical and simulated results of the spiral trajectory in the *x*-direction and *z*-direction are presented in Figure 13a,b, respectively. It can be seen from Figure 13 a that with the increase in the helix radius, the absolute error tends to increase, while the relative error fluctuates between 0.033% and 0.038%, as shown in Figure 13b. It can be inferred that for the spiral trajectory, the relative error of the movement is within 0.05% when the maximum radius of the movement area of the center point of the mobile platform does not exceed 5 × 10^−4^ m.

### 5.2. Computational Simulations of Spatial Pointing Trajectory

**Figure 14:**
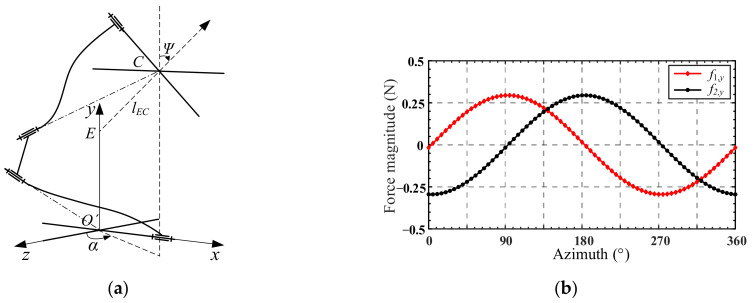
(**a**) Spatial pointing of mechanism; (**b**) curves of input forces.

This section continues the verification of the kinetostatic model through an example of a spatial pointing trajectory. As shown in Figure 14a, the spatial pointing of the mechanism is represented by the normal vector *l_EC_* of the mobile platform plane, where point *C* is the center of the mobile platform and point *E* is the intersection of the normal line of the mobile platform plane and the *y*-axis. By defining the included angle between the projection of vector *l_EC_* on the *O*′*xz* plane and the *z*-axis as the azimuth *α*, and the included angle between vector *l_EC_* and *y*-axis as the pitch *Ψ*, then the spatial pointing of the mechanism can be expressed as (*α*, *Ψ*) [28].

Given in the example is a set of spatial pointing trajectories, where the azimuth *α* takes 72 points at equal intervals within the range of [0, 360°], and the pitch angle *Ψ* is 0.025° (in the calculation process, the angle is in radian system). First, we convert the spatial pointing (the azimuth *α* and the pitch angle *Ψ*) into RPY (the Roll-Pitch-Yaw representation method of rotation around the *x-*, *y-*, and *z*-axes of the fixed coordinate frame) angles *θ_x_* and *θ_z_,* which rotates around the fixed *x*-axis and *z*-axis, respectively. A method of converting the spatially pointing RPY angle is provided in Appendix A.3. Then the angular displacements *θ_x_* and *θ_z_* of the 72 points were successively substituted in Equation (26) to obtained the input forces *f*_1,*y*_ and *f*_2,*y*_, the curves of which are shown in Figure 14b.

(26) $$\left[\begin{array}{c} \theta_{x}\\ \theta_{z} \end{array}\right]=\left[\begin{array}{c} \begin{array}{cc} C_{\theta_{x},f_{1,y}} & C_{\theta_{x},f_{2,y}}\\ C_{\theta_{z},f_{1,y}} & C_{\theta_{z},f_{2y}} \end{array} \end{array}\right]\left[\begin{array}{c} f_{1,y}\\ f_{2,y} \end{array}\right]=\left[\begin{array}{c} \begin{array}{cc} {\left(C_{TOF_{1}}\right)}_{\left[rows1,3,col5\right]} & {\left(C_{TOF_{2}}\right)}_{\left[rows1,3,col5\right]} \end{array} \end{array}\right]\left[\begin{array}{c} f_{1,y}\\ f_{2,y} \end{array}\right]$$

We define the matrix consisting of $C_{\theta_{x},f_{1,y}}$,$C_{\theta_{x},f_{2,y}}$,$C_{\theta_{z},f_{1,y}}$ and $C_{\theta_{z},f_{2y}}$ as the rotation mapping matrix, in which $C_{\theta_{x},f_{1,y}}$ represents the angular displacement of the mobile platform around the *x*-direction caused by a force along the *y*-direction at *F*_1_.

By employing the obtained input forces *f*_1,*y*_ and *f*_2,*y*_ in the Finite Element Analysis, the angular displacement of the mobile platform can then be obtained. It should be noted that the angle obtained from the finite element analysis is the RPY angle rotating around the fixed coordinate frame, and the RPY angle also needs to be converted into the azimuth *α* and pitch *Ψ*. A conversion method is given in Appendix A.4.

**Figure 15:**
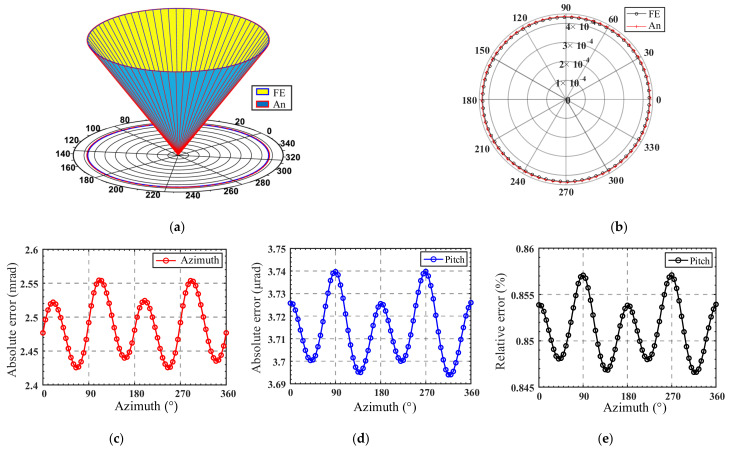
(**a**) Comparison between analytical and FE results of spatial pointing; (**b**) comparison between analytical and FE results of azimuth and pitch; (**c**) absolute error of azimuth; (**d**) absolute error of pitch; (**e**) relative error of pitch.

As shown in Figure 15a, the inner cone and outer cone respectively represent the FE-results and analytical results of the spatial pointing trajectory. The pitch is enlarged 1650 times for easier observation, and each generatrix on the cone represents a set of spatial points. To quantify the deviation between the spatial pointing azimuth *α* and pitch *Ψ* between the theoretical calculation results and the finite element analysis, Figure 15b is given, where the polar diameter represents the pitch of spatial pointing, and the polar angle represents the azimuth angle. The absolute error and relative error of the azimuth angle are shown in Figure 15c,d, respectively. The relative error of pitch is also given in Figure 15e.

As shown in Figure 15, the analytical results of spatial pointing show good consistency with the FE result. The maximum absolute errors of the azimuth and pitch are 2.56 mrad and 3.74 µrad, respectively, and the relative errors of pitch fluctuate between 0.845% and 0.86%, which verifies the accuracy of the kinetostatic model. It can be inferred that for the spatial pointing trajectory, the relative error of pitch is within 1% when the pitch angle *Ψ* does not exceed 0.025°. Meanwhile, it is observed that the errors caused by the kinetostatic model have a certain regularity with the change in the azimuth angle. Thus, the errors can be compensated by the control at the mobile platform to achieve the desired spatial pointing.

### 5.3. Influence of Parameters on Mapping Matrix

Section 5.1 and Section 5.2 verified the accuracy of the kinetostatic model. In this section, the 4-4R mechanism is employed as an object to analyze the effect of the flexure hinge structure parameter variations on the mapping matrix in Equations (25) and (26). The effect of the number of branches of the *n*-4R compliant parallel pointing mechanism on the mapping matrix is also explored. For convenience, the translational and rotational mapping matrices are defined as ***C****_T_* and ***C****_R_*, respectively.

**Figure 16:**
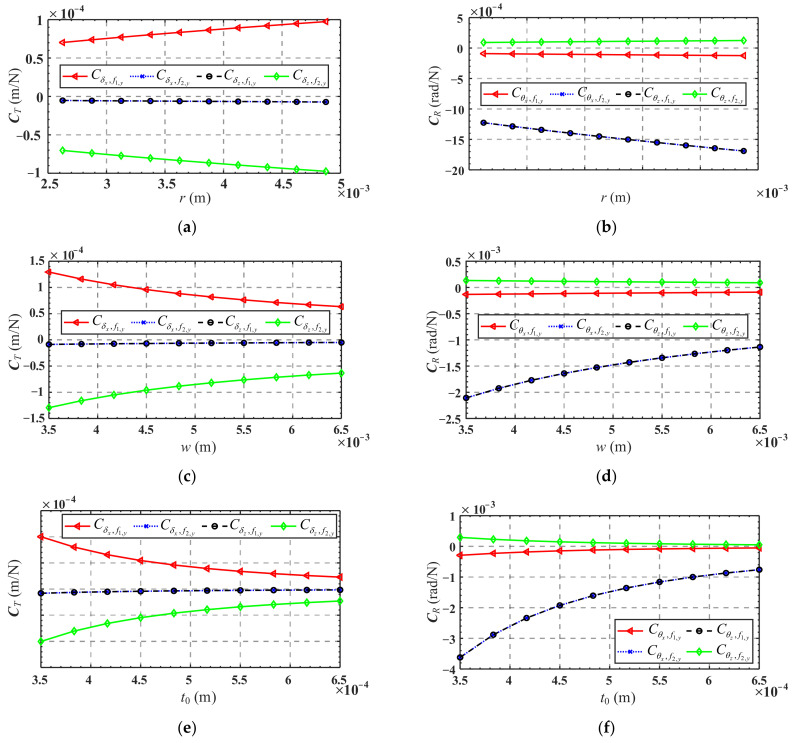
Variation of mapping matrix ***C**_T_* and ***C****_R_* in terms of flexure hinge structure parameters: (**a**) variation of ***C****_T_* in terms of the parameter *r*; (**b**) variation of ***C****_R_* in terms of the parameter *r*; (**c**) variation of ***C****_T_* in terms of the parameter *w*; (**d**) variation of ***C****_R_* in terms of the parameter *w*; (**e**) variation of ***C****_T_* in terms of the parameter *t*_0_; (**f**) variation of ***C****_R_* in terms of the parameter *t*_0_.

Figure 16a,c,e show the variations of mapping matrix *C_T_* in terms of the radius *r*, width *w*, and minimum thickness *t*_0_ of the flexure hinge, respectively, while the variations of mapping matrix ***C**_R_* in terms of these parameters are presented in Figure 16b,d,f, respectively.

It can be seen from Figure 16a,c,e that $C_{\delta_{x},f_{2,y}}$ and $C_{\delta_{z},f_{1,y}}$ are less affected by the structure parameters of the flexure hinge, while the absolute values of $C_{\delta_{x},f_{1,y}}$ and $C_{\delta_{z},f_{2,y}}$ increase as *r* increases and decrease as *w* and *t*_0_ decrease. It is indicated that when only the forces *f*_1,*y*_ or *f*_2,*y*_ are applied, increasing *r* will cause a larger linear displacement of the mobile platform center, while increasing *w* or *t*_0_ will yield a smaller linear displacement. As shown in Figure 16b,d,f, $C_{\theta_{x},f_{1,y}}$ and $C_{\theta_{z},f_{2,y}}$ are less affected by the structure parameters of the flexure hinge, and the absolute values of $C_{\theta_{x},f_{2,y}}$ and $C_{\theta_{z},f_{1,y}}$ increase as *r* increases and decreases as *w* or *t*_0_ increase. It is revealed that when only forces *f*_1,*y*_ or *f*_2,*y*_ are applied, increasing *r* will result in a larger angular displacement of the mobile platform center, and increasing *w* or *t*_0_ will cause a smaller angular displacement.

**Figure 17:**
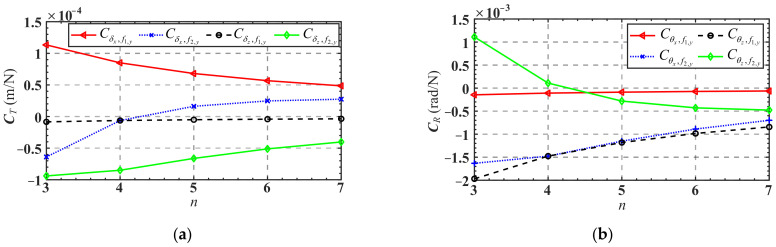
(**a**) Variation of mapping matrix ***C****_T_* in terms of branches number *n*; (**b**) variation of mapping matrix ***C****_R_* in terms of branches number *n*.

Figure 17 shows the variation of the translation mapping matrix ***C****_T_* and the rotation mapping matrix ***C****_R_* in terms of the branches number *n*. As can be seen from Figure 17a, with the increase in the branches number *n*, the absolute values of $C_{\delta_{x},f_{1,y}}$, $C_{\delta_{z},f_{1,y}}$, and $C_{\delta_{z},f_{2y}}$ decrease, while the absolute values of $C_{\delta_{x},f_{2,y}}$ decrease to 0 first and then gradually increase. Another interesting phenomenon is that when the branch number *n* increased from 4 to 5, the value of $C_{\delta_{x},f_{2,y}}$ changed from negative to positive. It indicates that when the number of the mechanism’s branches is less than or equal to 4, only applying a force *f*_2,*y*_ at *F*_2_ along the positive direction of the *y*-axis will cause a displacement of the mobile platform center along the negative direction of the *x*-axis, while the opposite conclusion is drawn when the branch number is more than or equal to 5.

As can be seen from Figure 17b, with the increase in the branch number *n*, the absolute values of $C_{\theta_{x},f_{1,y}}$, $C_{\theta_{x},f_{2,y}}$ and $C_{\theta_{z},f_{1,y}}$ decrease, while the absolute values of $C_{\theta_{z},f_{2,y}}$ decrease to 0 first and then gradually increases. There is a phenomenon similar to that shown in Figure 17a in that when the branches number *n* increased from 4 to 5, the value of $C_{\theta_{z},f_{2y}}$ changed from positive to negative. It suggests that when the number of the mechanism’s branches is less than or equal to 4, only applying a force *f*_2,*y*_ at *F*_2_ along the positive direction of the *y*-axis will cause an angular displacement of the mobile platform along the positive direction of the *z*-axis, while the opposite conclusion is drawn when the number of the mechanism’s branches is more than or equal to 5.

The influence of the structure parameters of the flexure hinge and the number of mechanism’s branches on the mapping matrix was discussed above. However, in practical applications, the loading force may be designed at different locations, thus also affecting the mapping matrix. Due to the limitation of space, this influence will not be discussed, and the reader can explore it in depth if interested.

## 6. Conclusions

In this paper, a novel class of *n*-4R compliant parallel micro pointing mechanisms is proposed and analyzed as follows: (1) The compliance model of the *n*-4R compliant parallel pointing mechanism is established by using coordinate transformation, and correctness of the compliance model is validated by finite element analysis. Then, the influence of the structure parameters of the flexure hinge, the scale parameters of the mechanism, and the branch number on the overall compliance of the mechanism are analyzed, which provides a reference for the compliance design of the *n*-4R compliant pointing mechanism. (2) Based on the mechanism compliance model and Hooke’s law, the elastic deformation governing equation of the equivalent spring system of the mechanism is derived, and the mapping relationship between the input force and the output displacement of a class of *n*-4R compliant parallel pointing mechanisms, i.e., the kinetostatic model, is established. (3) The kinetostatic model of the *n*-4R compliant parallel pointing mechanism is verified by finite element analysis through two given trajectories. The results show that the maximum relative errors of the analytical and FE-results of the two examples are 0.038% and 0.857%, respectively. Good consistency between the analytical and FE-results verifies the accuracy of the kinetostatic model, which lays a good foundation for the kinematic control of the mechanism. (4) The effects of the structure parameters of the flexure hinge and the number of branches on the mapping matrix in the kinetostatic model are also analyzed.

## Figures and Tables

**Table 1:** Parameters of translation transformation and rotation transformation.

Adjoint Transformation Matrix	*x*	*y*	*z*	*α*	*β*	*γ*
$Ad_{O1}^{O}$	$-r\cos\frac{\varphi }{2}$	$r\sin\frac{\varphi }{2}-L$	R	0	0	$\pi -\frac{\varphi }{2}$
$Ad_{O2}^{O}$	$r\sin\frac{\varphi }{2}-l$	$l\tan\frac{\varphi }{2}+r\cos\frac{\varphi }{2}-L$	0	$-\frac{\pi }{2}$	$\frac{\left(\varphi -\pi \right)}{2}$	0
$Ad_{O3}^{O}$	$-r\sin\frac{\varphi }{2}-l$	$r\cos\frac{\varphi }{2}-l\tan\frac{\varphi }{2}$	0	$-\frac{\pi }{2}$	$-\frac{\left(\varphi +\pi \right)}{2}$	0
$Ad_{O4}^{O}$	$r\cos\frac{\varphi }{2}$	$r\sin\frac{\varphi }{2}$	−R	0	0	$\frac{\varphi }{2}$

**Table 2:** Structural parameters of 4-4R compliant parallel pointing mechanism and loading position of ***F***_1_.

Parameters	Values (m)	Parameters	Values (m)	Parameters	Values (m)
*L*	0.1	*t*_0_	0.0005	*d*_1_	0.041
*R*	0.066	*r*	0.00375	*d*_2_	0.088
*l*	0.0666	*w*	0.005	*d*_3_	0.059
*φ*	60°				

**Table 3:** The comparison of analytical results and the FE-results of compliance.

Compliance		An	FE	Relative Errors
$C_{\theta_{x},m_{x}}$	(rad/Nm)	0.07303302	0.07241229	0.86%
$C_{\theta_{y},m_{y}}$	(rad/Nm)	0.00299648	0.00319900	6.33%
$C_{\theta_{z},m_{z}}$	(rad/Nm)	0.07303302	0.07241056	0.86%
$C_{\delta_{x},f_{x}}$	(m/N)	0.00021239	0.00021215	0.11%
$C_{\delta_{y},f_{y}}$	(m/N)	0.00005264	0.00005503	4.34%
$C_{\delta_{z},f_{z}}$	(m/N)	0.00021232	0.00021215	0.08%

**Table 4:** Variation range of geometric parameters of mechanism.

Parameters	Min (mm)	Max (mm)	Parameters	Min (mm)	Max (mm)
*L*	70	130	*r*	2.5	5
*l*	45	85	*t*_0_	0.35	0.65
*R*	45	85	*w*	3.5	6.5

## Data Availability

Not applicable.

## Appendix A

### Appendix A.1. Compliance Matrix of Right-Circular Flexure Hinge

The elements of the compliance matrix ***C*** of the right-circular flexure hinge are defined as follows [24]:

(A1) $$\left\{\begin{array}{c} C_{\theta_{z},f_{y}}=\frac{12}{Ew}\int_{0}^{2r}\frac{xdx}{t{\left(x\right)}^{3}}\\ C_{\theta_{z},m_{z}}=\frac{12}{Ew}\int_{0}^{2r}\frac{dx}{t{\left(x\right)}^{3}}\\ C_{\delta_{x},f_{x}}=\frac{1}{Ew}\int_{0}^{2r}\frac{dx}{t\left(x\right)}\\ C_{\delta_{y},f_{y}}=\frac{12}{Ew}\int_{0}^{2r}\frac{x^{2}dx}{t{\left(x\right)}^{3}}\\ C_{\delta_{y},m_{z}}=C_{\theta_{z},f_{y}}\\ C_{\delta_{z},f_{z}}=\frac{12}{Ew^{3}}\int_{0}^{2r}\frac{x^{2}dx}{t\left(x\right)}+\frac{1}{Gw}\int_{0}^{2r}\frac{dx}{t\left(x\right)}\\ C_{\delta_{z},m_{y}}=-\frac{12}{Ew^{3}}\int_{0}^{2r}\frac{xdx}{t\left(x\right)}\\ C_{\theta_{y},f_{z}}=C_{\delta_{z},m_{y}}\\ C_{\theta_{y},m_{y}}=\frac{12}{Ew^{3}}\int_{0}^{2r}\frac{dx}{t\left(x\right)} \end{array}\right.$$

where $t\left(x\right)=t_{0}+2r-2\sqrt{x\left(2r-x\right)}$, *E* is Young’s modulus of the hinge’s material, *G* is the shear modulus of the hinge’s material, *w* is the width of the flexure hinge, *r* is the radius of the flexure hinge, and *t*_0_ is the minimum thickness of the flexure hinge. $C_{\theta_{x},m_{x}}$ is determined by Equation (3.46) in Reference [26].

### Appendix A.2. Analytical Results and FE-Results of the Compliance Matrix of 3-4R and 5-4R Compliant Parallel Pointing Mechanisms

Analytical results and FE-results of the compliance matrix of 3-4R and 5-4R compliant parallel pointing mechanisms are as follows:

$C_{3-4R}^{\mathrm{An}}=\left[\begin{array}{cccccc} 9.74\times 10^{-2} & 0 & 0 & 1.89\times 10^{-5} & 0 & 4.87\times 10^{-3}\\ 0 & 4.00\times 10^{-3} & 0 & 0 & 1.22\times 10^{-4} & 0\\ 0 & 0 & 9.74\times 10^{-2} & -4.87\times 10^{-3} & 0 & 1.89\times 10^{-5}\\ 1.89\times 10^{-5} & 0 & -4.87\times 10^{-3} & 2.83\times 10^{-4} & 0 & 0\\ 0 & 1.22\times 10^{-4} & 0 & 0 & 7.02\times 10^{-5} & 0\\ 4.87\times 10^{-3} & 0 & 1.89\times 10^{-5} & 0 & 0 & 2.83\times 10^{-4} \end{array}\right]$	$C_{3-4R}^{\mathrm{FEM}}=\left[\begin{array}{cccccc} 9.65\times 10^{-2} & 0 & 0 & 2.03\times 10^{-5} & 0 & 4.83\times 10^{-3}\\ 0 & 4.27\times 10^{-3} & 0 & 0 & 1.31\times 10^{-4} & 0\\ 0 & 0 & 9.66\times 10^{-2} & -4.83\times 10^{-3} & 0 & 2.02\times 10^{-5}\\ 2.03\times 10^{-5} & 0 & -4.83\times 10^{-3} & 2.83\times 10^{-4} & 0 & 0\\ 0 & 1.31\times 10^{-4} & 0 & 0 & 7.34\times 10^{-5} & 0\\ 4.83\times 10^{-3} & 0 & 2.02\times 10^{-5} & 0 & 0 & 2.83\times 10^{-4} \end{array}\right]$
$C_{5-4R}^{\mathrm{An}}=\left[\begin{array}{cccccc} 5.84\times 10^{-2} & 0 & 0 & 1.13\times 10^{-5} & 0 & 2.92\times 10^{-3}\\ 0 & 2.40\times 10^{-3} & 0 & 0 & 7.30\times 10^{-5} & 0\\ 0 & 0 & 5.84\times 10^{-2} & -2.92\times 10^{-3} & 0 & 1.13\times 10^{-5}\\ 1.13\times 10^{-5} & 0 & -2.92\times 10^{-3} & 1.70\times 10^{-4} & 0 & 0\\ 0 & 7.30\times 10^{-5} & 0 & 0 & 4.21\times 10^{-5} & 0\\ 2.92\times 10^{-3} & 0 & 1.13\times 10^{-5} & 0 & 0 & 1.70\times 10^{-4} \end{array}\right]$	$C_{5-4R}^{\mathrm{FEM}}=\left[\begin{array}{cccccc} 5.79\times 10^{-2} & 0 & 0 & 1.22\times 10^{-5} & 0 & 2.90\times 10^{-3}\\ 0 & 2.56\times 10^{-3} & 0 & 0 & 7.89\times 10^{-5} & 0\\ 0 & 0 & 5.79\times 10^{-2} & -2.90\times 10^{-3} & 0 & 1.22\times 10^{-5}\\ 1.22\times 10^{-5} & 0 & -2.90\times 10^{-3} & 1.70\times 10^{-4} & 0 & 0\\ 0 & 7.89\times 10^{-5} & 0 & 0 & 4.40\times 10^{-5} & 0\\ 2.90\times 10^{-3} & 0 & 1.22\times 10^{-5} & 0 & 0 & 1.70\times 10^{-4} \end{array}\right]$

### Appendix A.3. Azimuth and Pitch Converted to RPY Angle

Assuming that the azimuth and pitch of the given mechanism are *α* and *Ψ,* respectively, the angular displacement *θ_x_* and *θ_z_* around the *x*-axis and *z*-axis can be obtained by:

(A2) $$\mathrm{rotz}(\theta_{z})\cdot \mathrm{rotx}(\theta_{x})\cdot \eta =\mathrm{roty}(\alpha )\cdot \mathrm{rotx}(\Psi )\cdot \eta ,\;\alpha ,\Psi \in \left[-\frac{\pi }{2},\frac{\pi }{2}\right]$$

where *η* denotes the pointing vector of the mobile platform center at the initial position of the *n*-4R compliant parallel pointing mechanism, *η* = [0, 1, 0]^T^.

### Appendix A.4. RPY Angle Converted to Azimuth and Pitch

Assuming that the angular displacements of rotation about the *x*-axis and *z*-axis obtained by simulation are *θ_x_* and *θ_z_*, the azimuth *α* and pitch *Ψ* of the mechanism can be obtained by:

(A3) $$\left\{\begin{array}{cc} {\left[\begin{array}{ccc} a_{1} & a_{2} & a_{3} \end{array}\right]}^{T} & =\;\mathrm{rotz}(\theta_{z})\cdot \mathrm{rotx}(\theta_{x})\cdot \eta \\ \alpha & =\;\arctan2(a_{1},a_{3})\\ \Psi & =\;\arctan\left(\frac{\sqrt{a_{1}^{2}+a_{3}^{2}}}{a_{2}}\right) \end{array}\right.$$

where *η* denotes the pointing vector of the mobile platform center at the initial position of the *n*-4R compliant parallel pointing mechanism, *η* = [0, 1, 0]^T^.
